# Diode Laser Surgery as a Conservative Management of Hairy Tongue Lesion Resistance to Treatment

**DOI:** 10.1155/2021/5656884

**Published:** 2021-12-14

**Authors:** Nazanin Samiei, Hadi Kaseb Ghane, Reza Fekrazad

**Affiliations:** ^1^Periodontics Department, School of Dentistry, Tehran University of Medical Sciences, Tehran, Iran; ^2^Department of Prosthodontics, Dental Branch, Islamic Azad University of Medical Sciences, Tehran, Iran; ^3^Radiation Sciences Research Center, Laser Research Center in Medical Sciences, AJA University of Medical Sciences, Tehran, Iran; ^4^International Network for Photo Medicine and Photo Dynamic Therapy (INPMPDT), Universal Scientific Education and Research Network (USERN), Tehran, Iran

## Abstract

Hairy tongue is a furry appearance on the dorsum surface of the tongue with variable colors. This lesion is due to defective shedding of filiform papillae. Various treatments are proposed for this condition like keratolytic agents, surgical procedure with a scalpel, or laser application. In this case study, we reported successful treatment of hairy tongue with a diode laser. The diode laser with wavelength 810 nm, power of 4 watts, a pulse width of 20 milliseconds, and an interval of 20 milliseconds by a 400-micron optical fiber was selected for treatment of this lesion. There was no bleeding during this minimally invasive surgery, and the patient experienced a low level of pain. Loss of taste function was completely resolved. Based on the result of this study, a diode laser can be a good alternative for minimally invasive surgical treatment of hairy tongue.

## 1. Introduction

Hairy tongue (HT), known as furred tongue, is referred to as an acquired benign tongue pathology. From the clinical view, HT is diagnosed through “hair-like” projections, developed when elongated, and hypertrophied filiform papillae are present on anterior 2/3 of dorsum aspect of the tongue. This pathology is the consequence of decreasing in cell desquamation and accumulation of keratinized layers [1].

HT may occur in various colors ranging from unpigmented, whitish, yellowish, bluish, tan, brown to black. Accordingly, what determines the color is an extrinsic factor that adheres to the lingual surface [2].

It may be prevalent within a wide range of 8.3 to 57% percent. Although there is no age limit for HT, its incidence and prevalence are positively correlated with age. It is reported to be more prevalent among men, yet females are also prone to this pathology [3]. According to a large cross-sectional study, an overall prevalence of HT was 11.3% with increased rates in men (18%) compared to women (6%) [4].

HT is associated with the following:1—poor oral hygiene; 2—smoking; 3—drugs (antibiotics such as penicillin, tetracycline, doxycycline, and erythromycin and medications inducing xerostomia including antidepressant, anticholinergics, and other drugs like olanzapine, aureomycin, and neomycin); 4—low fiber diets; 5—Candida albicans; 6—anemia; 7—alcohol; 8—dentures; 9—therapeutic radiation of head and neck. Scarcely has HT been found in human immunodeficiency virus (HIV) infection, graft versus host disease (GVHD), and internal malignancies [3].

It is asymptomatic and self-resolving in general, but patients may occasionally complain about halitosis, abnormal taste, gagging and tickling sensation, nausea, dysgeusia, and burning mouth sensation [5]. As the first-line treatment, it is necessary to identify and eliminate/modify predisposing factors; then, it comes to regularly brushing the tongue (gentle debridement with a toothbrush or tongue scraper) to promote desquamation and ultimately improve the oral hygiene [6].

Literature has also outlined the second-line therapy in different measures like topical retinoid, keratolytic agents (podophyllin), antimicrobial therapies, topical triamcinolone acetonide, gentian violet, salicylic acid, vitamin B complex, thymol, topical urea solution, and keratolytic agents like trichloroacetic acid. What merits attention is that the above-mentioned therapeutic measures are endowed with potential side effects from local irritation and possible systemic absorption. Surgical treatment of several oral lesions (namely, hemangioma, pyogenic granuloma, fibroma, and HT) can be addressed by laser application with different modalities like CO_2_, Erbium family, Nd: YAG, and diode lasers [7, 8]. Less invasive nature along with excellent hemostasis, wound disinfection, and great patient comfort make it more popular than a conventional scalpel in the surgical treatment of oral lesions [9]. Meserendio and Pick in 1995 [10] first introduced diode laser in oral surgery. Since then, diode laser has become popular due to its small size, low cost, and user-friendly features. In this case study, we are reporting the treatment of hairy tongue by diode laser and explain different aspects of this treatment modality.

## 2. Case Presentation

**Figure 1:**
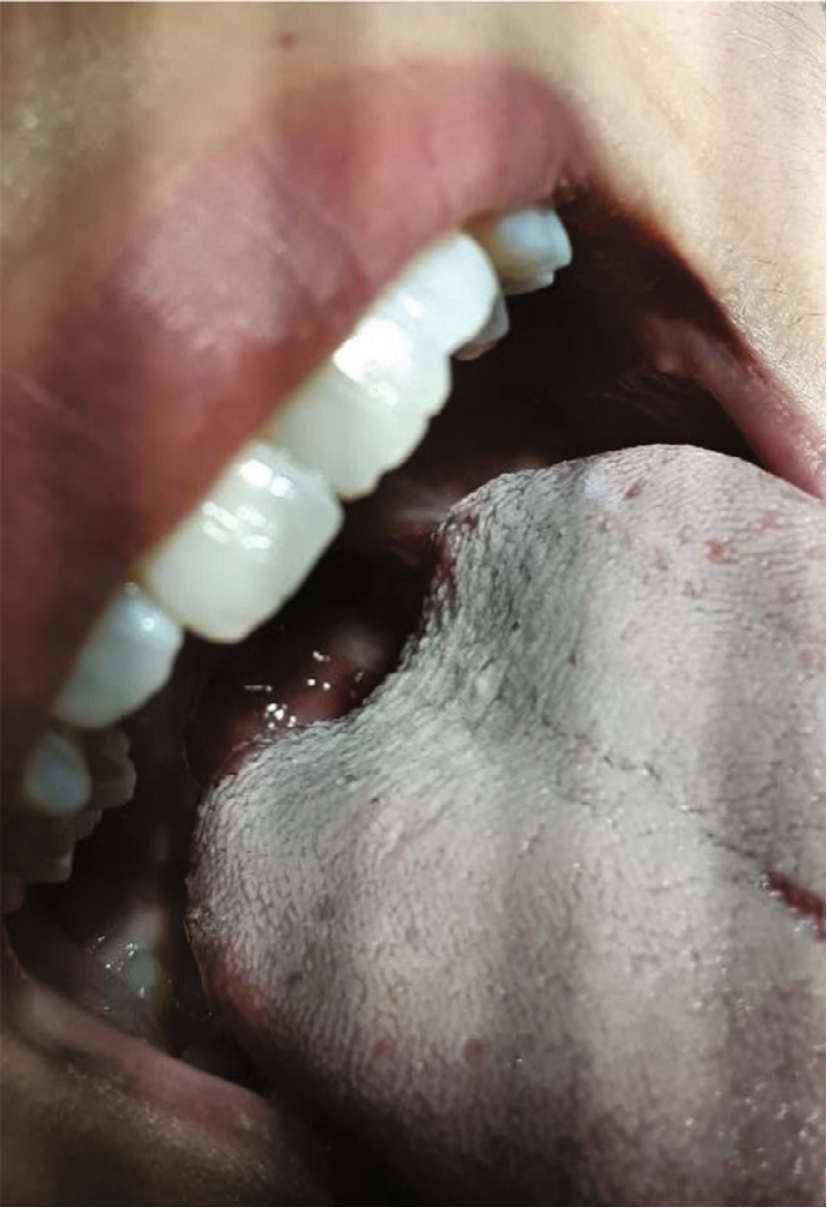
Dorsum of the tongue before treatment.

A 22-year-old nonsmoker woman presented with whitish-yellowish discoloration on the dorsum of the tongue and was referred to a private dental office (Figure 1). The patient mentioned that with about 1.5 year of evolution, she had experienced ageusia. She denied any other symptoms, namely, tongue pain and halitosis. She had been prescribed different medications and treatments, but no perceived improvement was noticed. This issue affected her quality of life and made her frustrated.

Medical history was unremarkable. She did not take any medication. She mentioned that she had high-stress lifestyle due to her job. Intraoral examination showed good oral hygiene according to Silness and Löe [11] plaque index. On soft tissue examination, no abnormalities were detected on the buccal and labial mucosa and palate. The whitish-yellowish discoloration appeared as an elongation of the filiform papillae on the dorsum of the tongue. As it could not be wiped by gauze, the case was clinically diagnosed with HT.

**Figure 2:**
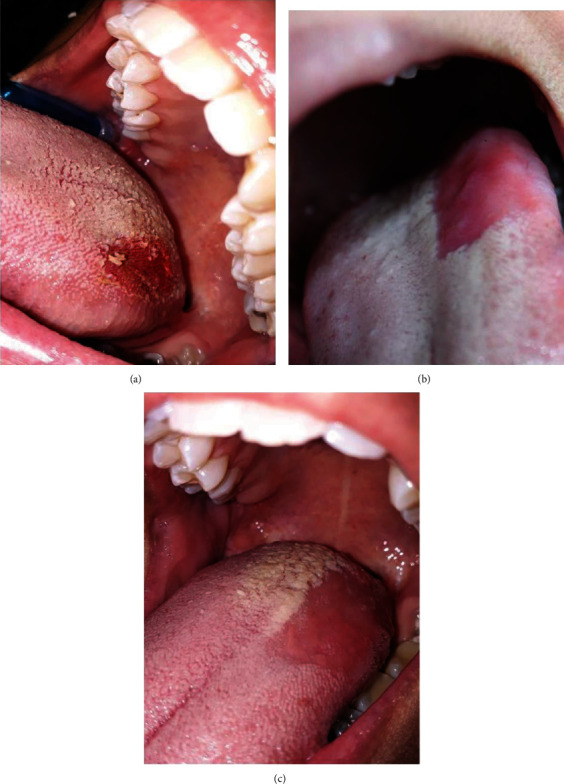
Clinical view of the tongue: (a) immediately after removing half of the lesion by the application of a diode laser; (b) one week after surgery; (c) two weeks after surgery.

After obtaining informed consent, a surgery was performed under local anesthesia (lidocaine HCL 2% with epinephrine 1 : 100,000). A diode laser (Twilight, Biolase Technologies, USA) was used by an expert surgeon. The laser enjoyed a wavelength of 810 nm, a power of 4 watts, a pulse width 20 milliseconds, and an interval of 20 milliseconds by a 400-micron optical fiber. The half of the lesion was removed by the laser in a slowly sweeping motion 4 mm above the surface and with a 30-degree angle relative to the tongue (to decrease penetration depth and reduce tissue damage) for 3-4 minutes, accompanied by high vacuum suction removal (Figure 2(a)). Special care was taken to remove only elongated filiform papillae and not to damage fungiform, circumvallate, and foliate papillae. Then, the surgical site was swept off with wet gauze. Chlorhexidine mouthwash (0.2%) was prescribed without any antibiotic therapies. No bleeding was reported after surgery. The patient experienced a low level of postoperative pain and just took few analgesics.

**Figure 3:**
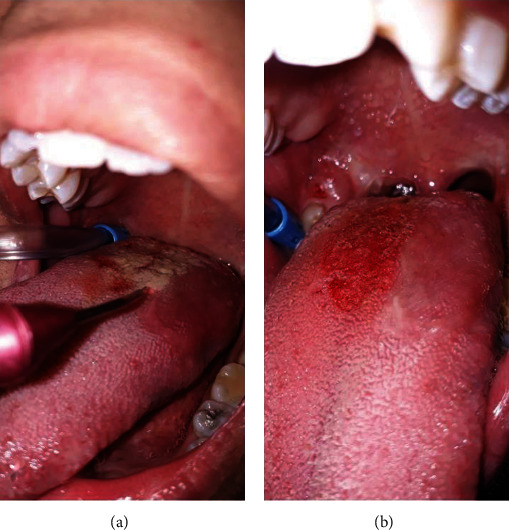
Clinical view of the tongue: (a, b) removing the other half of the lesion by the same diode laser mode.

**Figure 4:**
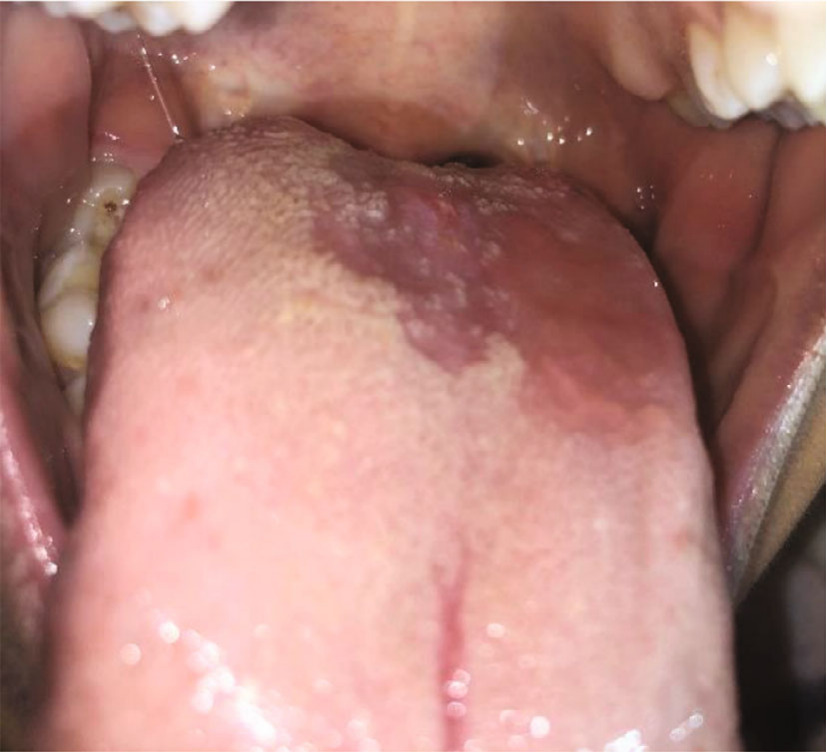
Three months after surgical procedure with a diode laser.

In the follow-up session after 1 and 2 weeks, partial healing was observed (Figures 2(b) and 2(c)). The other half of the lesion was removed by the same diode laser mode (Figures 3(a) and 3(b)). This procedure was done conservatively in two sessions in order to check the results in the follow-up sessions. After 1 month, complete healing was achieved with the resolution of ageusia. The patient was also examined 3 months later for assessing any sign of recurrence (Figure 4).

## 3. Discussion

HT is a relatively common abnormal coating on the dorsal surface of the tongue. This condition stems from elongation and lack of desquamation of filiform papillae, so they grow longer rather than falling off [12].

Various techniques have been employed to treat this lesion. We started with the second-line treatment (surgical procedure) as the patient's oral hygiene was good, and she did not have any risk factors (the requirements of the first-line treatment). Applying a knife to carry out the surgical excision of the papillae may be followed by some bleeding, where suturing is difficult and needs special skill. Also, it causes the removal of massive amount of tissue (with no control), which in turn increases the healing period and levels of pain [13]. Laser technology could provide an escape from these complications. In this case, we could use different laser modalities. Due to low penetration depth and high absorption, CO_2_ and Erbium family lasers are good options [14], but they are relatively high cost and not easily available [15]. The diode laser (450-1100) has become very popular in general dentistry as it is small in size and less costly, it enjoys fiber optic delivery, and it is user-friendly for minor oral soft tissues surgeries [16]. Regarding diode laser, research has shown that the wavelength around 810 nm is one of the most versatile wavelength ranges with regard to the number of different treatments it can be used for (soft tissue surgery, periodontal therapy, implantology, endodontics, photobiomodulation therapy, and teeth whitening) [17].

Thanks to the hemostasis and coagulation effect of the laser, the operator had an outstanding field visibility during the procedure and there was no need for suturing. Patients undergoing a diode laser surgery in the oral cavity seem to have less pain. In addition, it was proven that the diode laser can eliminate bacteria and disinfect the wound [18–20]; in this regard, there is no need for postsurgical antibiotics. Despite electrocutter and scalpel, the penetration depth of diode lasers can be adjusted; accordingly, it reduces tissue injury and improves wound healing.

In this study, the patient was satisfied with the result of the surgery and her ageusia was completely resolved. Consequently, her quality of life was significantly improved. To evaluate the lesion recurrence, she was examined 3 months later and there was no sign of recurrence. For further evaluation, long-term follow-ups were recommended.

## 4. Conclusion

Within the context of this study, application of the diode laser in the treatment of hairy tongue can be beneficial for both patients and clinicians. This modality provides minimal postoperative complications and a simple surgical procedure with the least possible side effects.
